# Beneficial Effect on Exercise Tolerance of a Comprehensive Rehabilitation Program in Elderly Obese Patients Affected With Heart Disease

**DOI:** 10.3389/fcvm.2021.652921

**Published:** 2021-06-08

**Authors:** Luca Alessandro Gondoni, Ferruccio Nibbio, Annamaria Titon

**Affiliations:** Unit of Cardiac Rehabilitation, Ospedale San Giuseppe, Piancavallo, Istituto Auxologico Italiano, Milan, Italy

**Keywords:** rehabilitation, obesity, elderly, coronary artery disease, heart failiure

## Abstract

**Aims:** The number of elderly patients affected with multiple chronic diseases is constantly increasing. Even though multiple studies demonstrated a beneficial effect of cardiac rehabilitation, we do not have data on the outcomes in elderly patients with obesity and heart disease.

**Methods:** We studied 772 consecutive obese subjects (275 women; 35.6%) aged ≥70 years, affected with coronary artery disease and/or heart failure. We conducted a symptom limited exercise test at the beginning and at the end of the program, which consisted of aerobic and strength physical activity, diet, and psychological counseling.

**Results:** Mean body mass index (BMI) at baseline was 37.6 ± 4.4 kg/m^2^ and decreased to 36.4 ± 4.3 kg/m^2^ (*P* < 0.001). At baseline, attained metabolic equivalents (METs) were 4.7 ± 1.7, and by the end of the program, they were 5.6 ± 2.1 (*P* < 0.001). The mean improvement was 21.6 ± 21.7% (median, 17.6%; 95% CI, 20.0–23.1%). Patients over 80 years old had similar results compared to the younger ones. Diabetics did worse than non-diabetic patients: the improvement they reached was 19.4 ± 18.9% vs. 23.8 ± 23.9% (*P* = 0.005). The presence of heart failure was significantly related to both the baseline and final performance, but the attained improvement was significantly greater in heart failure patients: 24.3 ± 23.8% vs. 16.3 ± 15.4% (*P* < 0.001). No patient had adverse events related to the program.

**Conclusion:** This study documents a significant improvement in exercise capacity in elderly obese patients affected with heart disease who underwent a rehabilitation program.

## Introduction

Elderly patients represent a critical issue in contemporary medicine. Aging is a cause of disability in itself, since all organs and systems lose functionality over the years, and this is particularly true if we consider patients affected with chronic diseases such as obesity and cardiopathy who have a high prevalence of disability (1). Moreover, we have to consider that both the number of elderly subjects and the prevalence of obesity are constantly increasing (2), which accounts for the considerable figures we have to deal with. Elderly patients are at a higher risk for complications and physical deconditioning: we know that physical activity is an important contributor to the prevention of disability and favors a healthy process of aging; moreover, exercise tolerance is also a very strong predictor of survival and is an easily available outcome of rehabilitation programs (3–5).

Multiple studies have demonstrated the beneficial effects of cardiac rehabilitation and exercise therapy, which are both considered keystones of secondary prevention, but unfortunately, a significant part of the available data pertains to young patients, while older patients are underrepresented in such programs, both for patient- and clinician-related issues. Several studies included elderly people and have long since established the benefit of rehabilitation in the elderly subset of patients, but those studies were conducted considering subjects aged over 65 as already “old” (6, 7). Rehabilitation in elderly persons could be a useful instrument to tackle the problems that such patients face, since they quite often have multiple comorbidities with progressive physical deconditioning and are therefore in particular need of comprehensive rehabilitation programs. We also have to consider that, currently, these programs are often restricted to people who have suffered an acute event, whereas our program, directed to patients with a complex clinical situation, is also addressed to subjects without an index event in agreement with the Italian Rehabilitation Guidelines endorsed by the Ministry of Health (8). Lastly, only few studies specifically tackled the issue of rehabilitation in the obese subset of elderly subjects, and, as a consequence, little is known about the results of a rehabilitation program in obese patients in their 70s or older who are affected with coronary artery disease (CAD) and/or heart failure (HF).

We therefore studied a group of patients aged over 70 years, who underwent a functional evaluation at the beginning and at the end of an in-hospital rehabilitation program, aiming at documenting the improvement in resistance to physical exertion.

## Patients and Methods

We retrospectively studied 772 consecutive obese white subjects (275 women, 35.6%) aged ≥70 years and affected with CAD and/or HF who were referred to San Giuseppe Hospital (Piancavallo, Italy) from 2002 to 2019 to undergo a short program of in-hospital comprehensive rehabilitation. The average length of stay was 24.9 ± 3.7 days (range, 14–36; median, 26 days). All patients gave informed written consent before exercise stress test and for the participation in the program. Every patient was in clinically stable conditions, and we excluded patients with recent (<1 month) acute events; to avoid selection bias, we included all the patients who did both the initial and the final exercise stress tests. For the purpose of our study, CAD was defined as prior myocardial infarction, coronary angioplasty, or coronary artery by-pass, while HF was defined as an ejection fraction (EF) <40% and/or symptoms (breathlessness, ankle swelling, and/or fatigue). Patients with normal or mildly reduced EF were considered affected with preserved EF HF (HFpEF) according to the European Society of Cardiology guidelines (9).

Body weight was measured in the morning after overnight fasting and voiding with a Wunder electronic scale; height was measured barefoot with a Seca 216 wall-mounted stadiometer. Body mass index (BMI) was calculated by dividing weight in kilograms by height^2^ in meters, and obesity was defined as body mass index (BMI) ≥30 kg/m^2^. In accordance with the World Health Organization classification, the patients were divided on the basis of their BMI as having mild obesity (30–34.9), moderate obesity (35–39.9), or severe obesity (≥40)^1^.

### Exercise Test

A symptom-limited exercise stress test was conducted during the morning on the second day of hospital stay and was repeated at the end of the rehabilitation program. Patients took their usual medications and had a light breakfast before the test. GE series 2000 motorized treadmill and Case ECG instrumentation (GE Medical Systems, Milwaukee, Wisconsin USA). We used a ramp protocol, tailored to the patients' characteristics, which has been described before (10). The reasons for test termination were limiting symptoms (fatigue, angina, dyspnea, muscular pain) or abnormal ECG (i.e., ventricular tachycardia, atrio-ventricular block, ST downsloping >2 mm, ST upsloping >1 mm). We measured the intensity of exercise using metabolic equivalents (METs). One MET represents the amount of oxygen consumed at rest and is equal to approximately 3.5 ml O_2_ kg^−1^ min^−1^. We derived our estimate of METs from treadmill speed and grade according to the formula METs = [speed (km/h) × 43.1 × (0.1 + 1.8 × grade + 3.5)/3.5].

### Other Tests

Each patient underwent an echocardiogram GE Vivid 7 instrument (GE Medical Systems, Milwaukee, Wisconsin USA) for the calculation of EF and a 24-h Holter ECG (ELA Medical - Sorin Group Milano Italia) to check for the presence of atrial fibrillation; mean 24-h heart rate was also measured.

### Physical Activity Program

All patients underwent a personal interview with an experienced physical trainer to tailor the activity program: the intensity of the program was determined on the basis of the baseline exercise test. The program entailed daily sessions (6 days a week) of aerobic activity, which included 30-min sessions of cyclo- or arm-ergometer and walking at low speed for about 45 min (3–4 METs). Patients performed also mild strength exercises that consisted of postural exercises and various free-body exercises. Patients were monitored for HR and arrhythmias during indoor activity. Target HR was measured on the basis of HR behavior during the baseline exercise test using the formula: target HR = baseline HR + 70% HR increase during effort. If patients did not exceed target HR and were feeling subjectively well, the workload was gradually increased. The compliance of the patients to the activity program was excellent.

### Diet

Resting energy expenditure (REE) was estimated by the Harris–Benedict equation (11). Diet was assigned by a specialist after a personal interview with the patient: the caloric intake was set at approximately 90% of REE. Periodically, on the basis of the amount of weight loss and of the patient's condition, the diet was checked and adapted. The hypocaloric diet derived 50% of energy from carbohydrates, 30% from lipids, and 20% from proteins.

### Other Interventions

Each patient was offered psychological evaluation, counseling, and support (when needed). Educational meetings on various topics were proposed on a weekly basis.

### Statistical Analysis

Continuous variables are described as mean ± SD. Median, 95% confidence intervals, and range were also reported whenever appropriate. Discrete variables are described as number and percentage. The difference between baseline and discharge values was calculated with a Student's *t-*test for paired data, and one-way ANOVA was used to compare the results of various dichotomous variables. Bivariate correlation and chi-square test were used when appropriate.

Statistical analysis has been conducted with IBM SPSS 26 package (IBM Corp., Armonk, NY, USA).

## Results

Our population consisted of 772 obese patients affected with multiple heart and systemic conditions (described in Table 1). Mean age was 74.2 ± 3.3 years (median, 73.5; range, 70.0–86.7).

**Table 1:** General characteristics of patients.

**Diagnosis**	**Mild obesity 240**	**Moderate obesity 335**	**Severe obesity 197**	**Overall 772**
Coronary artery disease	205 (85.4%)	281 (83.9%)	137 (69.5%)	623 (80.7%)
Heart failure	133 (55.4%)	223 (66.6%)	155 (78.7%)	511 (66.2%)
Hypertension	181 (75.4%)	264 (78.8%)	178 (90.4%)	629 (81.5%)
Valvular heart disease	24 (10.0%)	52 (15.5%)	30 (15.2%)	116 (15.0%)
Diabetes	124 (51.7%)	178 (53.1%)	86 (43.7%)	388 (50.3%)

The pharmacological treatment is described in Table 2.

**Table 2:** Pharmacological treatment.

**Treatment**	**Number**	**%**
Beta-blockers	567	73.4
Diuretics	526	68.1
ACE-inhibitors	405	52.5
AT2 receptor blockers	306	39.6
Calcium antagonists	261	32.8
Antiplatelet	617	79.9
Antiarrhythmics	90	11.7
TNG	72	9.3
Ranolazine	22	2.8
Doxazosin	133	17.2
Clonidine	11	1.4
Statins and/or Ezetimibe	693	89.8
Insulin	192	24.8
Other antidiabetics	260	33.7

*ACE, angiotensin-converting enzyme; AT, angiotensin; TNG, nitro-glycerine*.

Mean BMI at baseline was 37.6 ± 4.4 kg/m^2^ (range, 30.0–53.7), and it was significantly reduced at the end of the program when it had lowered to 36.4 ± 4.3 kg/m^2^ (*P* < 0.001). The mean improvement was 3.15 ± 1.6% (median, 3.1; 95% CI, 3.04–3.26%). The absolute weight decrease was 3.2 ± 1.7 kg. Only 12 patients did not lose weight.

EF was reduced (≤40%) in 100 subjects and mildly reduced (40–55%) in 350 subjects. It was normal (>55%) in the remaining 322 patients. Among the patients with heart failure, the percentage of those with HFpHF was 75%.

At baseline, attained METs were 4.7 ± 1.7. When considering the ratio between attained and expected METs, we found our patients had reached 81 ± 30% of the predicted value (12, 13). By the end of the program, the exercise test was repeated, and the results were, respectively, 5.6 ± 2.1 and 97 ± 35% (*P* < 0.001 for both). The improvement, expressed as percentage, was 21.6 ± 21.7% (median, 17.6%; 95% CI, 20.0–23.1%). Several patients reached a very low workload, as defined by a cutoff of 3.5 METs (14). At the initial test, 244 patients (31.6%) did not exercise beyond that cutoff; the number was almost halved at the discharge test, and only 124 (16.1%) reached a value below 3.5 METs. A borderline, yet significant, difference was evident when we considered the duration of the program: using the median value (26 days) as a cutoff, we found that the 368 patients whose stay was <26 days improved their exercise capacity less than the 404 patients whose stay was ≥26 days, having reached, respectively, an improvement of 20 ± 20% vs. 23 ± 22% (*P* = 0.046).

In our population, only 58 subjects were current smokers, 442 were ex-smokers, and 273 had never smoked. We found no significant difference in attained METs at the baseline test and in METs improvement among the three categories.

Baseline exercise capacity, either expressed by absolute values or by ratio between predicted and expected, was negatively influenced by diuretic treatment (4.4 ± 1.6 vs. 5.3 ± 1.8 METs; 77 ± 29% vs. 88 ± 30%). On the contrary, no drug regimen affected the attained improvement.

We also analyzed the outcomes in several subgroups of patients. Forty-eight patients were over 80 years old, and they achieved an improvement that was identical to the less old ones.

Diabetic patients, even though their metabolic parameters were bettered (data not shown), did worse as compared to the non-diabetic patients: the improvement that they were able to reach was 19.4 ± 18.9% vs. 23.8 ± 23.9% (*P* = 0.005); baseline characteristic did not differ from their non-diabetic counterpart with the exception of a higher number of female patients who represented 41% of diabetic and 30% of non-diabetic patients; insulin treatment did not worsen the outcome.

The presence of HF, either with reduced or preserved EF, was significantly related to both the baseline and final performance: as expected, patients with HF did worse than the ones without HF; at baseline, the attained METs were, respectively, 3.9 ± 1.2 and 6.3 ± 1.5 METs, while at discharge, the values were 4.8 ± 1.5 and 7.3 ± 1.9 (*P* < 0.001 for both comparisons). Interestingly, however, the improvement in HF patients was significantly greater: 24.3 ± 23.8% vs. 16.3 ± 15.4% (*P* < 0.001). Patients with atrial fibrillation had more frequently a diagnosis of HF, but they obtained the same improvement as their counterpart without fibrillation. Higher heart rate at 24-h ECG was significantly related to both baseline and discharge test (the higher the heart rate, the lower the exercise capacity) but not to tolerance improvement. The correlation factors between mean 24-h HR and exercise tolerance were −0.140 and −0.152, respectively, for the baseline and final test (*P* < 0.001 for both), while it was only −0.014 (*P* = 0.698) for the improvement.

We also divided the outcome in tertiles, and we identified several variables that influenced the results. The tertiles identified those with a poor result (<12% improvement in exercise capacity), an intermediate result (13–26% of improvement), and a good result (>26% improvement). A good performance at baseline was related to a lower improvement, and the ones who had a longer hospital stay did better. The results are described in Table 3.

**Table 3:** Factors influencing the outcome.

**Variable**	**Tertiles of outcome**	**Mean**	**SD**	**95% CI**		***P***
Hospital Stay	≤12%	24.39	3.93	23.91	24.87	0.006
	13–26%	24.91	3.77	24.45	25.37	
	>26%	25.44	3.44	25.02	25.86	
Baseline attained METs	≤12%	4.99	1.81	4.77	5.22	<0.001
	13–26%	4.85	1.66	4.65	5.06	
	>26%	4.26	1.64	4.06	4.46	
Age	≤12%	74.3	3.2	73.9	74.7	0.670
	13–26%	74.2	3.3	73.7	74.6	
	>26%	74.1	3.2	73.7	74.5	
Baseline BMI	≤12%	37.6	4.2	37.1	38.1	0.532
	13–26%	37.9	4.4	37.3	38.4	
	>26%	37.4	4.4	36.9	38.0	
Baseline weight	≤12%	100.0	13.7	98.3	101.	0.221
	13–26%	102.1	15.4	100.1	103.9	
	>26%	101.9	15.7	100.0	103.8	
EF	≤12%	0.53	0.10	0.51	0.54	0.099
	13–26%	0.53	0.09	0.52	0.54	
	>26%	0.52	0.10	0.50	0.53	

*BMI, body mass index; EF, left ventricular ejection fraction*.

Women were more prevalent among the ones who did worse (*P* = 0.020). In addition, considering the 89 patients who did not improve their exercise performance, women were more prevalent: 42 (15.3%) were women and 47 (9.4%) were men (*P* = 0.015).

## Discussion

Our study attests to a significant improvement in exercise capacity in elderly obese patients affected with heart disease who underwent a short rehabilitation program. No patient experienced any adverse event that could be related to the activity program.

While considering that a home-based program has similar results and is less expensive than a hospital-based one (15), we chose the latter because, in our experience, it offers both patients and clinicians better opportunities (16) such as visiting the patient every day and by so finely optimizing pharmacological treatment (17), increasing exercise level safely in a strictly supervised environment beyond the values that the patient could reach on their own, and eventually offering nutritional, psychological, and educational support with a positive effect on their well-being (18). The benefits of a hospital-based program are even more clear-cut in fragile people who are often poorly compliant to any kind of prescription. We also found that the longer the duration of hospital stay is, the greater the improvement in the capacity of exercising becomes. Lastly, the hospital program overcomes one of the barriers that hinder participation in rehabilitation, i.e., the distance that patients have to travel from their home: our patients come from almost every region of Italy, since ours is one of the few programs specifically designed for obese heart patients.

As we know, age is the main determinant of exercise tolerance, and all the formulae that are used to predict exercise capacity are based mainly on age and sex. Since also obesity has a significant impact on exercise capacity (10), it is not surprising that our patients reached quite a low peak effort. We chose to concentrate our attention on exercise capacity because of its high prognostic value and its impact on subjective well-being. As known since the late 1980s, in healthy people, one can say that the fitter they are, the longer they will survive (19). Such perspective has been confirmed also in the obese population establishing the so-called “fat but fit” condition (20). Even though the evidence for the metabolically healthy obese model only comes from observational studies, it seems to be trustworthy, and besides, the prognostic role of exercise tolerance in elderly subjects has been amply demonstrated (21). Exercise workload at the end of a rehabilitation program has a significant prognostic value, and the patients who only reached 3.5 METs were at higher risk for future events (13): the great improvement we documented in our population could be seen as an encouraging outcome since both obesity and physical inactivity affects disease states and mortality rates.

A low level of physical fitness is associated with increased risk of all-cause and cardiovascular mortality. It has been shown already many years ago that patients who improve their physical fitness reduce their mortality risk and were less likely to die from all causes and from cardiovascular disease during follow-up than persistently unfit subjects (22). The improved exercise capacity could possibly have a positive feedback effect and make our patients less sedentary than they used to be: even that only point could be regarded as a very good gain. The improvement that we measured, even though we considered only exercise tolerance as outcome variable, could well be the effect of various factors that have a positive influence on exercise capacity: these can possibly include the improvement of heart rate and blood pressure parameters, the bettering of the psychological status, and the better adaptation to the treadmill. Nevertheless, whatever the reasons that stand behind the improvement, any increase in exercise tolerance exerts a positive effect on quality and duration of life.

Another important issue we have to deal with is the loss of muscle mass that elderly subjects very often face. In our program, we pay particular attention to exercise tolerance, rather than focusing on weight loss that may appear low in our population, because we know that weight loss obtained through dieting alone, using a very low-calorie diet, could cause a decrease in fat-free mass and could be detrimental to elderly patients.

We do not have follow-up data, and therefore, we do not exactly know what the impact of our program on the future of our patients will be: they may live longer, but there is concern that obesity could reverse many of the public health successes that have occurred in recent decades and could erode the overall health status of people. We have to say that our previous published data (23) were not satisfactory, and whether the improvement that we demonstrated might also have an impact on long-term survival has to be tested in follow-up studies, but the results could only be measured without the support of a control group. Even in the absence of long-term data, we do know from a previous study that there is a positive relationship between cardiac rehabilitation and long-term outcomes such as death and myocardial infarction (24). Anyway, lifespan is not the only issue at stake: contemporary medicine continues its pursuit of life extension, sometimes forgetting to consider the drawbacks of reaching such a goal. As a matter of fact, further life extension might expose the elderly to an elevated risk for age-related diseases, and in long-lived populations, a substantial part of life occurs when the risk for frailty and disability is dramatically high (25). Therefore, survival is not the only issue relevant to the elderly: control and reduction in disability might be even more important. According to the World Health Organization definition, “Healthy Aging” is the process of developing and maintaining the functional ability that enables well-being in older age, where by “functional ability” is meant “having the capabilities that enable people to be and do what they have reason to value.” Those capabilities include, among others, to meet their basic needs, to be mobile, and to have relationships. By implementing programs that are capable of improving exercise capacity, we can have an impact on the intrinsic capacity of the patient, i.e., on the physical capacities and possibly also on the mental capacity, including the ability to walk, think, see, hear, and remember.

It is quite obvious that older patients are often facing a complex clinical situation, and we are noticing a significant increase in the prevalence of disabling morbid conditions such as obesity: a vast proportion of the population participating in rehabilitation programs are obese (26). As Baltes and coworkers pointed out (27), successful aging is possible when latent reserve capacities have the opportunities to be empowered. Physical activity is certainly one of the most relevant examples and, contrary to cognitive functioning, sometimes falls beyond our control; it is trainable and therefore easier to preserve. According to this view, successful aging should be supported by adequate care, tailored to the needs of our elderly patients. Even though we quite often ignore the subjective patients' notion of the meaning of well-being, we can state that an increase in exercise capacity, which also means a greater autonomy in everyday activities, could be an important point to address.

Obesity is strongly associated with disability prevalence, and the excess risks of disability are greater than the excess risk of mortality due to obesity. This is sometimes brought into question by the so-called obesity paradox. The concept was reassessed in a review published in 2018, and the paradigm was tentatively shifted to the lean paradox, i.e., it is not the obese population who has a better prognosis but is the leaner part who has a worse prognosis (28). Instead, several conditions can explain the high prevalence of disability in the obese: among them we find diabetes, arthritis, gait disturbances, coronary heart disease, heart failure, and depression, and all of them are improved by rehabilitation. The fact that, even with a small decrease in BMI, our patients were able to reach a great improvement in exercise tolerance is a relevant issue, considering that fitness markedly improves life expectancy also in obese patients (29).

In our opinion, a relevant point in our research is offering rehabilitation to stable patients without a recent index event; this is an approach that has been largely underused but could be a very interesting option for selected patients: obesity is, by definition, a chronic disease and therefore deserves a different approach from other medical conditions (15). Collins et al. showed that positive effects on functional capacity were present when the program started within 3 months from an index event, therefore opening a space to patients who are similar to ours (30). As already stated, Italian Guidelines consider the option of intensive in-hospital rehabilitation for “Patients affected with non-surgical heart diseases at intermediate or high risk in whom the rehabilitation program, even though not strictly following an index event, could prevent clinical deterioration and disease progression.” Moreover, obese patients experience psychological distress levels that are higher compared to the non-obese subset of patients (31) and can therefore benefit from intensive rehabilitation programs even without any index event.

Diabetic patients did worse than non-diabetic patients. Several years ago, we documented a similar result (23) but in a younger population. The reasons whereby diabetic patients have a poorer outcome are not fully clear. The baseline characteristics of diabetic patients were similar to the ones of the non-diabetic population with the one exception of a slight, albeit significant, female prevalence in the diabetic population that could play a minor role considering that women did a little worse than men. Another possible explanation could be found in the autonomic disarray of diabetic patients, which has an impact on the autonomic regulation in those patients. Rehabilitation improves heart rate parameters in obese patients (32), but resting and peak heart rate have different behaviors in diabetic as compared to non-diabetic patients, since the latter tend to lower resting heart rate and increase peak effort heart rate more than their diabetic counterpart do (33).

The very elderly did as good as the younger subjects: this is good news and confirms the results of previous studies that dealt with outpatient rehabilitation (34, 35).

We think that another strength of our study is the use of a treadmill to test exercise capacity: many programs use the 6-min walking test or the 200-m fast walk test to measure the outcomes of a rehabilitation program, particularly in the older population such as ours. In our experience, however, exercise capacity measured using a treadmill exercise stress test is a much more accurate indicator of performance as compared to other tests, and only attained METs accurately predict survival in many subsets of patients. Safety concerns have been raised about subjecting fragile patients to a maximal exercise stress test. We have not experienced any unfavorable event, and we can therefore assure that, using an appropriate protocol, maximal exercise testing is safe also with the elderly subset of the population.

Lastly, it has to be noted that our protocol was conducted in a single center and represents the routine activity at our institution: it is therefore a non-randomized study in a real-life population.

A possible limit of our study may be the fact that we did not directly measure oxygen consumption: nevertheless, it should be remembered that in everyday practice in most rehabilitation programs, the outcome is evaluated by a normal exercise test, and we therefore preferred this simple, but widely used, instrument as a means for testing exercise tolerance.

Another limit could be ascribed to the absence of a control group, and this is one of the critical issues in rehabilitation research: when we are dealing with behavioral interventions, a randomized controlled trial poses several problems, since we do not really have the possibility to identify a placebo group. Just as an example, how can we tell some of our patients not to exercise since they have been allocated to a non-intervention group in a randomized trial? Therefore, we think that research should accept as a consolidated notion that comprehensive rehabilitation is effective in heart patients and should therefore focus on peculiar populations such as ours to identify potential subsets of patients who could benefit from rehabilitation (36).

In conclusion, a short in-hospital rehabilitation program favorably affected exercise tolerance in a cohort of obese elderly patients.

## Data Availability Statement

The raw data supporting the conclusions of this article will be made available by the authors, without undue reservation.

## Ethics Statement

Ethical review and approval was not required for the study on human participants in accordance with the local legislation and institutional requirements. The patients/participants provided their written informed consent to participate in this study.

## Author Contributions

All authors listed have made a substantial, direct and intellectual contribution to the work, and approved it for publication.

## Conflict of Interest

The authors declare that the research was conducted in the absence of any commercial or financial relationships that could be construed as a potential conflict of interest.

## Footnotes

^1^Available online at: https://www.euro.who.int/en/health-topics/disease-prevention/nutrition/a-healthy-lifestyle/body-mass-index-bmi

## References

[1] Keeney T Jette AM. Individual and environmental determinants of late-life community disability for persons ageing with cardiovascular disease. Am J Phys Med Rehabil. (2019) 98:30–4. 10.1097/PHM.0000000000001011 30095448 PMC6705120

[2] Chooi YC Ding C Magkos F. The epidemiology of obesity. Metabolism. (2019) 92:6–10. 10.1016/j.metabol.2018.09.005 30253139

[3] Snader CE Marwick TH Pashkow FJ Harvey SA Thomas JD Lauer MS. Importance of estimated functional capacity as a predictor of all-cause mortality among patients referred for exercise thallium single-photon emission computed tomography: report of 3400 patients from a single center. J Am Coll Cardiol. (1997) 30:641–8. 10.1016/S0735-1097(97)00217-9 9283520

[4] Brawner CA Al-Mallah MH Ehrman JK Qureshi WT Blaha MJ Keteyian SJ. Change in maximal exercise capacity is associated with survival in men and women. Mayo Clin Proc. (2017) 92:383–90. 10.1016/j.mayocp.2016.12.016 28185659

[5] Safdar B Mangi AA. Survival of the fittest: impact of cardiorespiratory fitness on outcomes in men and women with cardiovascular disease. Clin Ther. (2020) 42:385–92. 10.1016/j.clinthera.2020.01.014 32088022

[6] Lavie CJ Milani RV Littman AB. Benefits of cardiac rehabilitation and exercise training in secondary coronary prevention in the elderly. J Am Coll Cardiol. (1993) 22:678–83. 10.1016/0735-1097(93)90176-2 8354798

[7] Shapira I Fisman EZ Motro M Pines A Ben-Ari E Drory Y. Rehabilitation in older coronary patients. Am J Geriatr Cardiol. (1995) 4:48–55. 11416355

[8] Linee guida nazionali su cardiologia riabilitativa e prevenzione secondaria delle malattie cardiovascolari: sommario esecutivo a cura del Gruppo di Lavoro dell'Agenzia per i Servizi Sanitari Regionali dell'Istituto Superiore di Sanità – Piano Nazionale Linee Guida – e del Gruppo Italiano di Cardiologia Riabilitativa e Preventiva (GICR). G Ital Cardiol. (2008) 9:286–97. 10.1714/652.7614 18543799

[9] Ponikowski P Voors AA Anker SD Bueno H Cleland JGF Coats AJS. 2016 ESC Guidelines for the diagnosis and treatment of acute and chronic heart failure: The Task Force for the diagnosis and treatment of acute and chronic heart failure of the European Society of Cardiology (ESC)Developed with the special contribution of the Heart Failure Association (HFA) of the ESC. Eur Heart J. (2016) 37:2129–200. 10.1093/eurheartj/ehw128 27206819

[10] Gondoni LA Liuzzi A Titon AM Taronna O Nibbio F Ferrari P. A simple tool to predict exercise capacity in obese patients with ischemic heart disease. Heart. (2006) 92:899–904. 10.1136/hrt.2005.064758 16339818 PMC1860706

[11] Harris JA Benedict FG. A Biometric Study of Basal Metabolism in Man. Washington, DC: Carnegie Institution of Washington (1919).

[12] Gulati M Black HR Shaw LJ Arnsdorf MF Merz CN Lauer MS. The prognostic value of a nomogram for exercise capacity in women. N Engl J Med. (2005) 353:468–75. 10.1056/NEJMoa044154 16079370

[13] Morris CK Morrow K Froelicher VF Hideg A Hunter D Kawaguchi T. Prediction of cardiovascular death by means of clinical and exercise test variables in patients selected for cardiac catheterization. Am Heart J. (1993) 125:1717–26. 10.1016/0002-8703(93)90764-Z 8498316

[14] Brawner CA Abdul-Nour K Lewis B Schairer JR Modi SS Kerrigan DJ. Relationship between exercise workload during cardiac rehabilitation and outcomes in patients with coronary heart disease. Am J Cardiol. (2016) 117:1236–41. 10.1016/j.amjcard.2016.01.018 26897640

[15] Anderson L Sharp GA Norton RJ Dalal H Dean SG Jolly K. Home-based versus centre-based cardiac rehabilitation. Cochrane Database Syst Rev. (2017) 6:CD007130. 10.1002/14651858.CD007130.pub4 28665511 PMC6481471

[16] Capodaglio P Lafortuna C Petroni ML Salvadori A Gondoni L Castelnuovo G. Rationale for hospital-based rehabilitation in obesity with comorbidities. Eur J Phys Rehabil Med. (2013) 49:399–41. 23736902

[17] Goyal P Gorodeski EZ Marcum ZA Forman DE. Cardiac rehabilitation to optimize medication regimens in heart failure. Clin Geriatr Med. (2019) 35:549–60. 10.1016/j.cger.2019.06.001 31543185 PMC7233375

[18] Manzoni GM Villa V Compare A Castelnuovo G Nibbio F Titon AM. Short-term effects of a multi-disciplinary cardiac rehabilitation programme on psychological well-being, exercise capacity and weight in a sample of obese in-patients with coronary heart disease: a practice-level study. Psychol Health Med. (2011) 16:178–89. 10.1080/13548506.2010.542167 21328146

[19] Blair SN Kohl HW Paffenbarger RS Clark DG Cooper KH Gibbons LW. Physical fitness and all-cause mortality. A prospective study of healthy men and women. JAMA. (1989) 262:2395–401. 10.1001/jama.262.17.2395 2795824

[20] Ortega FB Lavie CJ Blair SN. Obesity and cardiovascular disease. Circ Res. (2016) 118:1752–70. 10.1161/CIRCRESAHA.115.306883 27230640

[21] Kokkinos P Myers J Faselis C Panagiotakos DB Doumas M Pittaras A. Exercise capacity and mortality in older men: a 20-year follow-up study. Circulation. (2010) 122:790–7. 10.1161/CIRCULATIONAHA.110.938852 20697029

[22] Blair SN Kohl HW Barlow CE Paffenbarger RS Gibbons LW Macera CA. Changes in physical fitness and all-cause mortality. A prospective study of healthy and unhealthy men. JAMA. (1995) 273:1093–8. 10.1001/jama.273.14.1093 7707596

[23] Gondoni LA Titon AM Nibbio F Caetani G Augello G Mian O. Short-term effects of a hypocaloric diet and a physical activity programme on weight loss and exercise capacity in obese subjects with chronic ischaemic heart disease: a study in everyday practice. Acta Cardiol. (2008) 63:153–9. 10.2143/AC.63.2.2029521 18468193

[24] Hammill BG Curtis LH Schulman KA Whellan DJ. Relationship between cardiac rehabilitation and long-term risks of death and myocardial infarction among elderly Medicare beneficiaries. Circulation. (2010) 121:63–70. 10.1161/CIRCULATIONAHA.109.876383 20026778 PMC2829871

[25] Olshansky SJ. From lifespan to healthspan. JAMA. (2018) 320:1323–4. 10.1001/jama.2018.12621 30242384

[26] Capodaglio P Ventura G Petroni ML Cau N Brunani A. Prevalence and burden of obesity in rehabilitation units in Italy: a survey. Eur J Phys Rehabil Med. (2019) 55:137–9. 10.23736/S1973-9087.18.05393-5 30156090

[27] Baltes PB Lindenberger U Staudinger UM. Life-span theory in developmental psychology. In: Lerner RM Damon W, editors. Handbook of Child Psychology. New York, NY: Wiley (2006). 10.1002/9780470147658

[28] Elagizi A Kachur S Lavie CJ Carbone S Pandey A Ortega FB. An overview and update on obesity and the obesity paradox in cardiovascular diseases. Prog Cardiovasc Dis. (2018) 61:142–50. 10.1016/j.pcad.2018.07.003 29981771

[29] Ortega FB Lee DC Katzmarzyk PT Ruiz JR Sui X Church TS. The intriguing metabolically healthy but obese phenotype: cardiovascular prognosis and role of fitness. Eur Heart J. (2013) 34:389–97. 10.1093/eurheartj/ehs174 22947612 PMC3561613

[30] Collins ZC Suskin N Aggarwal S Grace SL. Cardiac rehabilitation wait times and relation to patient outcomes. Eur J Phys Rehabil Med. (2015) 51:301–9. 25213305

[31] Terada T Chirico D Tulloch HE Scott K Doucet É Pipe AL. Psychosocial and cardiometabolic health of patients with differing body mass index completing cardiac rehabilitation. Can J Cardiol. (2019) 35:712–20. 10.1016/j.cjca.2019.02.024 31151706

[32] Gondoni LA Bertone G Titon AM Nibbio F Montano M. Improvement of chronotropic incompetence after a rehabilitation program in obese patients with coronary artery disease treated with beta-blockers: a practice level study. Phys Med Rehabil Int. (2018) 5:1153.

[33] Kim HJ Joo MC Noh SE Kim JH. Long-term outcomes of cardiac rehabilitation in diabetic and non-diabetic patients with myocardial infarction. Ann Rehabil Med. (2015) 39:853–62. 10.5535/arm.2015.39.6.853 26798598 PMC4720760

[34] Mehta H Sacrinty M Johnson D St Clair M Paladenech C Robinson K. Comparison of usefulness of secondary prevention of coronary disease in patients <80 versus ≥80 years of age. Am J Cardiol. (2013) 112:1099–103. 10.1016/j.amjcard.2013.05.058 23830528

[35] Bierbauer W Scholz U Bermudez T Debeer D Coch M Fleisch-Silvestri R. Improvements in exercise capacity of older adults during cardiac rehabilitation. Eur J Prev Cardiol. (2020) 27:1747–55. 10.1177/2047487320914736 32321285

[36] Gondoni LA. Chapter 7 cardiac rehabilitation. In: Capodaglio P Faintuch J Liuzzi A, editors. Disabling Obesity. Berlin; Heidelberg: Springer-Verlag (2013). 10.1007/978-3-642-35972-9

